# Cyanogenic millipede genome illuminates convergent evolution of cyanogenesis-related enzymes

**DOI:** 10.1371/journal.pgen.1011955

**Published:** 2025-11-24

**Authors:** Takuya Yamaguchi, Yasuhisa Asano

**Affiliations:** Biotechnology Research Center and Department of Biotechnology, Toyama Prefectural University, Imizu, Toyama, Japan; Universidad de Valparaiso, CHILE

## Abstract

Hydrogen cyanide (HCN) is a highly toxic biogenic compound. Unlike most natural defensive chemicals, which are typically lineage-specific, the biosynthesis and liberation of HCN, called “cyanogenesis”, occur sporadically among arthropod and plant lineages. This suggests that cyanogenesis has evolved independently numerous times in the animal and plant kingdoms. Although cyanogenesis was identified in millipedes 140 years ago, the cyanogenesis-related enzymes in these arthropods have not yet been fully identified. Here, we report a complete set of cyanogenesis-related enzymes in the millipede *Chamberlinius hualienensis* based on an analysis combining genome sequencing and biological characterisation. The gene encoding hydroxynitrile lyase, which catalyses the liberation of HCN from (*R*)-mandelonitrile, and its paralogous genes were clustered, indicating sequential duplication of their coding genes, giving rise to hydroxynitrile lyase in millipedes. We discovered that (*R*)-mandelonitrile cyanohydrin biosynthesis in *C. hualienensis* utilises a flavin-dependent monooxygenase (ChuaMOxS) for the initial aldoxime synthesis step, similar to the process in ferns, instead of cytochrome P450 (CYP) as in higher plants and insects. Although a single CYP is responsible for subsequently converting aldoxime into cyanohydrin in plants and insects, the reaction involves two enzymes in millipedes. We found two millipede CYPs (CYP4GL4 and CYP30008A2) that catalyse aldoxime dehydration to produce nitrile, in addition to CYP3201B1, which then catalyses the formation of (*R*)-mandelonitrile from nitrile. The discovery of cyanogenesis-related enzymes in millipedes demonstrates that cyanogenic millipedes evolved these enzymes independently from plants and insects, providing a deeper understanding of the mechanisms underlying the evolution of metabolic pathways.

## Author summary

The biosynthesis of natural defensive chemicals is usually lineage-specific; however, cyanogenesis (hydrogen cyanide biosynthesis) occurs sporadically among animal and plant lineages. This suggests that the cyanogenesis pathway has arisen numerous times in different kingdoms; however, examples of the independent evolution of the entire pathway are rare. By sequencing genome of the cyanogenic millipede *Chamberlinius hualienensis* and performing biochemical characterization of its enzymes, we identified the gene cluster containing the gene encoding the cyanide releasing enzyme hydroxynitrile lyase along with homologous genes. In addition, we discovered three previously unidentified enzymes responsible for the cyanohydrin biosynthesis. Cyanogenesis-related enzyme genes were specifically detected in cyanogenic millipede order (Polydesmida) but not in other non-cyanogenic millipede orders. Moreover, our identified enzymes exhibited no phylogenetic relationships with cyanogenesis-related enzymes from cyanogenic plants and insects. Collectively, our findings demonstrate that cyanogenic millipedes independently evolved cyanogenesis-related enzymes from plants and insects, providing a deeper understanding of the mechanisms underlying the evolution of metabolic pathways.

## Introduction

Chemical substances, so-called secondary or specialised metabolites, are involved in the most important biotic interactions between plants and their herbivores/pathogens and between animals and their predators/parasites [1]. Selection for increased fitness has resulted in each living organism synthesising a distinct set of specialised metabolites appropriate for its environment [2]. The acquisition of new enzymes with new functions in secondary metabolite biosynthesis is generally a result of divergent evolution. Thus, most specialised metabolites are typically lineage-specific. In contrast, convergent evolution has been reported, in which different lineages independently evolved the ability to synthesise identical specialised metabolites [1,3,4]. However, it is less common and less well understood than divergent evolution [5].

Many chemical substances produced by plants and animals play crucial roles in defence. Among the most deterring and toxic biogenic substances, hydrogen cyanide (HCN) inhibits mitochondrial cytochrome *c*-oxidase in the cellular respiration system, limiting the organism’s ability to use oxygen [6]. The biosynthesis and liberation of HCN, known as “cyanogenesis”, are widespread among plants [7]. Cyanogenic plants accumulate cyanogenic glycosides as stable cyanide precursors. When plant tissues are disrupted by herbivores or pathogens, glycosides are degraded by β-glycosidase and hydroxynitrile lyase (HNL) to release aldehydes or ketones and HCN via cyanohydrins [8].

**Fig 1:**
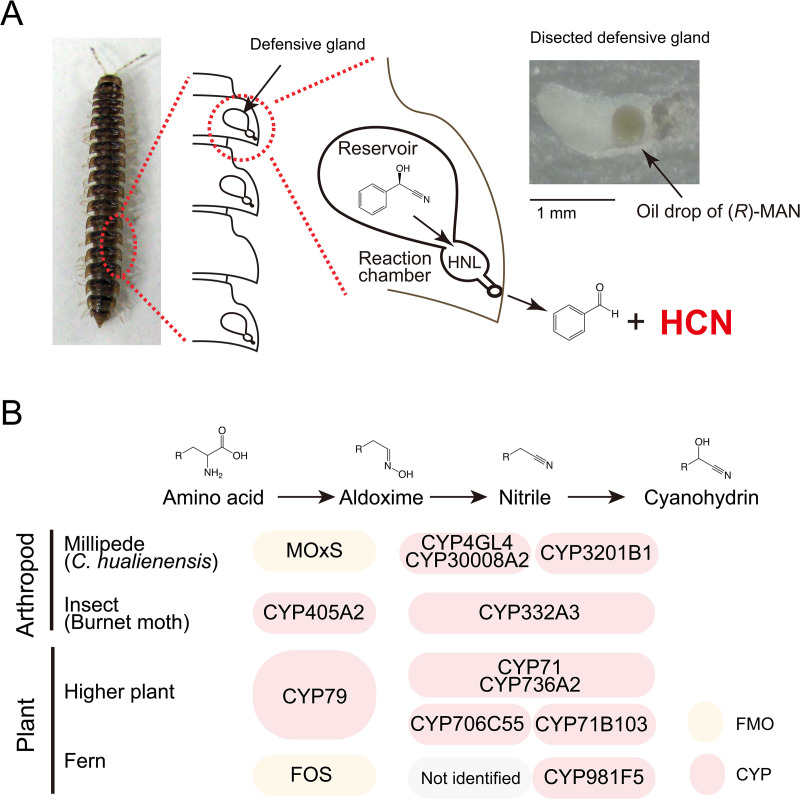
Schematic overview of cyanogenesis in *Chamberlinius hualienensis* and cyanohydrin biosynthesis in millipedes, insects, and plants. (A) *C. hualienensis* and a schematic view of cyanogenesis. Paranota of the millipede house defensive glands and consist of reservoir and reaction chamber. (*R*)-Mandelonitrile (MAN) stored in the reservoir is admitted to the reaction chamber, converted by hydroxynitrile lyase (HNL) to hydrogen cyanide (HCN) and benzaldehyde, and released to the outside via ozopores. (B) In arthropods (millipede and burnet moth) and plants, cyanohydrin is commonly synthesised from amino acids via aldoxime and nitrile. Different classes of flavin-dependent monooxygenases (FMO) and cytochrome P450s are responsible for the biosynthesis.

Cyanogenesis has been observed in several arthropods, including millipedes, mites, beetles, true bugs, and butterflies [9]. Cyanogenesis was first discovered in millipedes in 1882 [10]. Among millipedes, members of the order Polydesmida are generally cyanogenic [11] and they produce (*R*)-mandelonitrile (MAN) as a cyanide precursor and its related/decomposed compounds, such as benzaldehyde, benzyl alcohol, benzoyl cyanide, benzoic acid, mandelonitrile benzoate, and hydrogen peroxide [11–13]. Millipedes store (*R*)-MAN in the reservoirs of defensive glands housed in the paranota and eject cyanogenic secretions through small openings called ozopores located on the dorsal surface near the tips of paired notal projections. (Fig 1A) [14]. Despite being identified over 100 years ago, to our knowledge, enzymes associated with cyanogenesis have yet to be fully characterized in millipedes.

We selected the Polydesmida millipede *Chamberlinius hualienensis* Wang, which invaded Japan from Taiwan, as a model cyanogenic millipede. The millipede often forms large swarms [15], which is beneficial for research purposes but can cause problems in local communities because, as these swarms can enter houses and sometimes cause train delays [16]. We previously purified HNL (ChuaHNL) from 29 kg of *C. hualienensis* [17]. Although ChuaHNL shared no amino acid sequence similarity with other proteins [17], HNLs are conserved among cyanogenic millipedes [18,19]. Based on structural analyses, plant and microbial HNLs have been classified into six groups: glucose–methanol–choline (GMC) oxidoreductases, α/β-hydrolases, serine carboxypeptidases, cupins, Bet v1 proteins, dimeric α + β barrel folds, and Zn² ⁺ -dependent alcohol dehydrogenases. Millipede HNLs were newly assigned to the lipocalin superfamily, highlighting their distinct structural features and unique evolutionary origin [18,20,21]. HNL is involved in liberating HCN and benzaldehyde from (*R*)-MAN in millipedes (Fig 1A). It also catalyses the reverse reaction, namely, the asymmetric synthesis of chiral cyanohydrin, which is a valuable building block for the synthesis of pharmaceuticals from various aldehydes and cyanide sources [17]. ChuaHNL showed the highest specific activity (7420 U/mg) for (*R*)-MAN synthesis among the HNLs isolated from plants and microorganisms [17]. ChuaHNL also exhibited excellent enantioselectivity towards various substrates and high heat and pH stability. Optically pure cyanohydrins are valuable building blocks; therefore, millipede HNLs are used to produce valuable substances [18,20,22].

(*R*)-MAN is biosynthesised from l-phenylalanine (Phe) via (*E/Z*)-phenylacetaldoxime (PAOx) and phenylacetonitrile (PAN) in cyanogenic millipedes [23,24]. The sequence of biosynthesis to cyanohydrin from amino acid via aldoxime and nitrile corresponds to that of cyanohydrin biosynthesis in other cyanogenic plants and arthropods (Fig 1B). In higher plants, two multifunctional cytochrome P450 monooxygenases (CYPs), CYP79 and CYP71, are generally involved in cyanohydrin biosynthesis with several exceptions (Fig 1B) [25]. CYP79 catalyses the conversion of amino acids into the corresponding aldoximes, which are subsequently transformed into cyanohydrins via nitriles by CYP71. For example, in Japanese apricot and almond, CYP79D16 catalyses the conversion of l-Phe into PAOx, which is subsequently transformed into MAN via PAN by CYP71AN24 [26,27]. MAN is glucosylated by UGT85A and UGT94AF to produce amygdalin [27,28], which is a stable storage form of the cyanohydrin. In some fern species, flavin-dependent monooxygenase (FMO) named FOS1 produces aldoxime (Fig 1B) [29], and in *Arabidopsis thaliana*, an FMO named FOX1 is involved in the synthesis of a cyanogenic metabolite [30]. The burnet moth (*Zygaena filipendulae*) is the only arthropod for which a complete set of biosynthetic enzymes has been identified (that is, CYP405A2 and CYP332A3; Fig 1B) [31]. Higher plant and insect CYPs catalyse the same reactions but are not homologous, indicating convergent evolution. Compared to plants or burnet moths, *C. hualienensis* may utilise a different set of enzymes. CYP3201B1 from *C. hualienensis* synthesises (*R*)-MAN from PAN but not (*E/Z*)-PAOx [23]. Thus, the conversion of aldoxime to cyanohydrin is catalysed by a single CYP in most higher plants and burnet moths but by multiple enzymes in *C. hualienensis*. However, aldoxime- and nitrile-producing enzymes have not yet been identified in millipedes.

Given the scattered distribution of cyanogenesis and the phylogenetic distribution of millipedes, insects, and plants, millipedes independently evolved cyanogenesis-related enzymes from insects and plants while the pathway sequence was conserved. In this study, we aimed to identify the enzymes involved in the cyanogenesis pathway in the cyanogenic millipede *C. hualienensis* through genome sequencing analysis. We then biochemically characterised these enzymes.

## Results

### Sequencing, assembly, and annotation of the *C. hualienensis* genome

**Fig 2:**
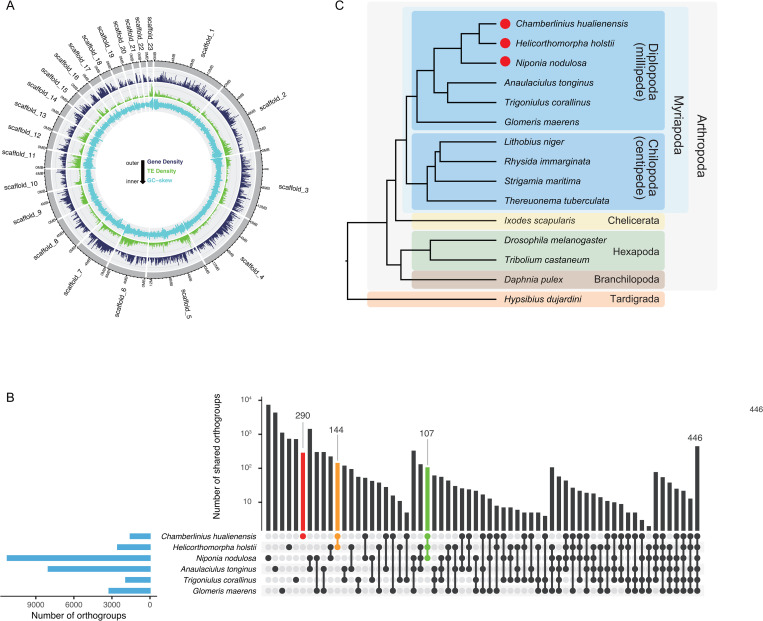
*C. hualienensis* genome annotation. (A) Characterisation of different elements on the millipede scaffolds. From outer to inner ring, the > 10 kb scaffolds of the assembled *C. hualienensis* genome, gene density, transposable element density, and GC-skew. (B) Upset plot comparing shared orthogroup in millipedes. (C) Phylogenetic tree. The tree is rooted with *Hypsibius dujardini* as the outgroup. Polydesmida species are labeled with red circles.

In total, 100.42 Gb (~700 × coverage) of clean data from Illumina and 20.04 Gb (~140 × coverage) of clean data from PacBio Sequel were obtained (S1 and S2 Tables). A schematic diagram of the hybrid assembly is shown in S1 Fig. The assembled *C. hualienensis* genome was 143,521,810 bp, with a scaffold N50 size of 12,989,427 bp (Fig 2A and Table 1), representing 99.3% of the estimated genome size (144.5 Mb) (S2 Fig). The integrity of the assembled genome was assessed. First, the short reads obtained from the Illumina sequencing data were compared with the assembled genome. The percentage of reads mapped to the draft genome was greater than 96%. Next, BUSCO [32] analysis revealed that 95.6% of the single-copy arthropod orthologues were complete. These results indicated that the *C. hualienensis* genome assembly was of high quality and coverage. The genome was the smallest among the genome-sequenced Myriapods, millipedes and centipedes, ranging from 181 to 2530 Mbp [33,34] (S3 Fig). As the size of repetitive sequences, genomic sequence comprised of transposable elements (TE) and simple repeats, are known to positively correlate with genome size [35], we analysed the TE in *C. hualienensis* genome. In the *C. hualienensis* genome assembly, 25.4 Mb of repetitive sequences, which cover 17.7% of the genome, were identified (S3 Table). The unclassified elements were the most abundant type of repetitive sequence in *C. hualienensis*, spanning 15.8 Mb (11.0%) of the genome. Class I long terminal repeat retrotransposons, class II DNA transposons, and non-long terminal repeat elements account for 1.55%, 3.26%, and 0.25%, respectively, of the genome. A strong correlation between transposable element content and genome size across *C. hualienensis* and other millipedes and centipedes genomes was observed (R^2^ = 0.98, *P* = 7.85e-08; regression line y = 128 + 1.63 x) (S3 Fig), in agreement with the earlier report [33].

**Table 1:** Assembly and annotation statistics of *Chamberlinius hualienensis* genome.

Assembly features	Statistics
BioProject Accession number	PRJDB13209
Estimate genome size (Mb)	144
Number of contigs	104
Contig N50 (Mb)	4.46
Number of scaffolds	55
Scaffold N50 (Mb)	13.0
Assembly size (bp)	143,521,810
GC content (%)	31.9
Complete ratio of BUSCO (%)	95.6
Repeat content (%)	17.7
Gene number	14132
Average gene length (bp)	5971
Average coding sequence length (bp)	1550

Ab initio gene prediction programs and evidence from RNA sequencing (S4 Table) were integrated to annotate the *C. hualienensis* genome. A total of 14,132 gene models were predicted for the genome (Table 1). BUSCO assessment indicated that the predicted genes covered 93.8% of arthropod-conserved orthologues. Among the predicted proteins, 12,443 (80.3%) were annotated using GenBank, 12,541 (81.2%) using interproscan, 11,702 (75.7%) using eggnog mapper, and 11,038 (71.4%) using pfam.

### Orthogroup and species tree

To infer the evolutionary history of *C. hualienensis*, orthorogroup analysis was performed to identify single-copy orthologous proteins. Across myriapod and other arthropod species: millipedes (*Helicorthomorpha holstii*, *Nipponia nodulosa*, *Anaulaciulus tonginus*, *Trigoniulus corallinus*, and *Glomeris maerens*), centipedes (*Rhysida immarginata*, *Lithobius niger*, *Thereuonema tuberculata*, and *Strigamia martima*), other arthropods (*Ixodes scapularis*, *Daphnia pulex*, *Tribolium castaneum*, and *Drosophila melanogaster*), and the tardigrade *Hypsibius dujardini*, 41,810 orthogroups were identified. In total, 3,104 orthogroups contained all species, 18,160 contained a subset of species, and 21,323 were species-specific orthogroups. Six millipede species shared 446 orthogroups, three Polydesmida millipede species shared 107 orthogroups, two cyanogenic millipedes shared 144 orthogroups, and *C. hualienensis* shared 290 species-specific orthogroups (Fig 2B). Phylogenetic reconstruction was conducted based on 102 single-copy orthologous proteins shared by the abovementioned organisms (Fig 2C). Three Polydesmida species, *C. hualienensis*, *H. holstii*, and *N. nodulosa,* formed a monophyletic clade (Fig 2C).

### *ChuaHNL* and its paralogous gene cluster on the genome

**Fig 3:**
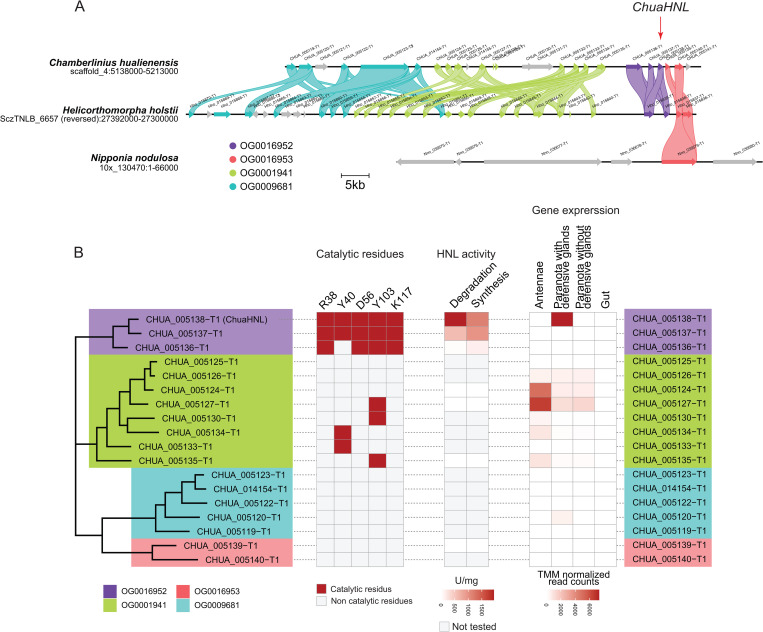
Gene cluster and characterisation of *ChuaHNL* and its paralogues. (A) *ChuaHNL* and its paralogue cluster on the *C. hualienensis* genome and synteny among Polydesmida millipedes. (B) Phylogenetic tree and catalytic residues, HNL activity, and gene expression (TMM-normalised reads counts) of *ChuaHNL* and its paralogues.

HNL is a key enzyme of cyanogenesis in millipedes. This enzyme catalyzes the release of hydrogen cyanide from (*R*)-MAN [36], which is accumulated in *C. hualienensis* approximately 570 µg and 596 µg per male and female millipede, respectively (S4 Fig). However, evolutionary origin of the enzyme remains unclear. We investigated whether *C. hualienensis* harbours *ChuaHNL* homologous genes (paralogues) by orthogroup analysis. *ChuaHNL* (CHUA_005138) and its two paralogues (CHUA_005137 and CHUA_005136) were grouped into the orthogroup OG0016952. These protein-coding genes were clustered in the genome (Fig 3A). CHUA_005137 and CHUA_005136 shared 70% and 54% amino acid sequence identities with ChuaHNL, respectively (S5 Fig), and shared all and four of the five catalytic residues [18,20,21], respectively (Figs 3B and S6). Further, seventeen *ChuaHNL*-like genes were observed within 75 kb of the *ChuaHNL* gene cluster (Fig 3A). The proteins encoded by these genes were assigned to orthogroups OG0001941, OG0009681, and OG0016953, unlike ChuaHNL, which belonged to OG0016952 (Fig 3A and 3B). However, the amino acid sequences of these proteins shared 11–21% identity at the amino acid level with ChuaHNL (S5 Fig) and eight structurally important Cys residues (S6 Fig), which formed inter- and intra-disulfide bonds in ChuaHNL [21], except *CHUA_005132*. This gene was partially truncated and is considered malfunctional (S6 Fig). These results suggested that ChuaHNL and the paralogous proteins, except for CHUA_005132, share lipocalin folds, although these paralogous proteins are grouped into four orthogroups. Orthologues of *ChuaHNL* and its paralogous genes were found in the *H. holstii* genome. They clustered in the *C. hualienensis* genome, and their syntheny was conserved (Fig 3A). The *N. nodulosa* genome only encoded an orthologous protein belonging to OG0016953, not the gene cluster in *C. hualienensis* and *H. holstii* (Fig 3A). Lipocalins are a group of widely distributed proteins that can be found in animals, plants, and bacteria [37]. Millipede HNLs and related proteins are phylogenetically distant from lipocalins of other arthropods, plants, and bacteria (S7 Fig), and their fold is distinct from plant and microbial HNLs (S8 Fig).

### Biochemical characterisation of ChuaHNL and its paralogous proteins

Whether ChuaHNL paralogous proteins exhibit HNL activity, we heterologously produced ChuaHNL and six paralogous proteins (CHUA_005137 and CHUA_005136 from OG0016952; CHUA_005139 from OG0014028; CHUA_005135, CHUA_005127, and CHUA_005124 from OG000814) as N-terminal His-tagged proteins using the yeast *Pichia pastoris* expression system [38]. These proteins were isolated from 120 mL of the culture supernatant using a nickel affinity column. SDS-PAGE showed that all proteins were secreted into the medium (S9 Fig). Among them, ChuaHNL, CHUA_005137, and CHUA_005136 catalysed (*R,S*)-MAN degradation, indicating that CHUA_005137 and CHUA_005136 are functional HNLs. However, the others did not exhibit HNL activity (Fig 3B), which agrees with the fact that these paralogous proteins did not have the catalytic residues of millipede HNLs (Figs 3B and S6). We attempted to create variants with full active site residues but were unable to do so in *P. pastoris*. To compare ChuaHNL and the paralogous proteins having HNL activity, recombinant proteins were purified from 1.5 L of culture supernatant (S5–S7 Tables). ChuaHNL and two paralogous proteins catalysed the degradation of racemic MAN into benzaldehyde and HCN with the following kinetic parameters (S10A Fig): ChuaHNL, *V*_max_ = 2950 ± 227 µmol/min/mg, *K*_m_ = 13.7 ± 2.35 mM; CHUA_005137, *V*_max_ = 916 ± 67.4 µmol/min/mg, *K*_m_ = 12.9 ± 2.16 mM; CHUA_005136 *V*_max_ = 50.0 ± 0.681 µmol/min/mg, *K*_m_ = 1.10 ± 0.0618 mM. ChuaHNL, CHUA_005137, and CHUA_005136 also catalysed its reverse reaction, namely the (*R*)-MAN synthetic reaction from benzaldehyde and potassium cyanide, with the following kinetic parameters (S10B Fig): ChuaHNL, *V*_max_ = 1310 ± 63.6 µmol/min/mg, *K*_m_ = 4.31 ± 0.771 mM; CHUA_005137 *V*_max_ = 1280 ± 80.1 µmol/min/mg, *K*_m_ = 4.50 ± 0.994 mM; CHUA_005136 *V*_max_ = 677 ± 93.6 µmol/min/mg, *K*_m_ = 2.36 ± 0.696 mM, *K*_i_ = 21.1 ± 5.74 mM. When the same activity of the enzyme was used for the reaction (that is, 8 U/mL), the enantiomeric excess of (*R*)-MAN produced was 92.1%, 91.5%, and 83.8%, respectively (S11 Fig). These results indicated that CHUA_005137 and CHUA_005136 have excellent *R*-selectivity in cyanohydrin synthesis, similar to ChuaHNL [17].

To elucidate the expression pattern of ChuaHNL and its paralogous genes in *C. hualienensis*, we performed RNA-seq analysis of four tissues, including the antennae, paranota-containing defensive glands, paranota without defensive glands, and gut (S8 Table). *ChuaHNL* was specifically expressed in the paranota-containing defensive glands (Fig 3B) in accordance with previous gene expression analyses [17]. CHUA_005137 and CHUA_005136 were expressed in traces, indicating that these proteins do not accumulate in the defensive glands, although they exhibit HNL activity. A few paralogous proteins, such as CHUA_005124 and CHUA_005127, were highly expressed in the antennae (Fig 3B). ChuaHNL belongs to the lipocalin family [21] and the lipocalin proteins often functions as a carrier protein for small hydrophobic molecules [37]. The expression of ChuaHNL paralogues in the antennae is consistent with that of lipocalin genes in the antennae of several insect species [39].

### CYPome and identification of CYPs involved in (*R*)-MAN biosynthesis

**Fig 4:**
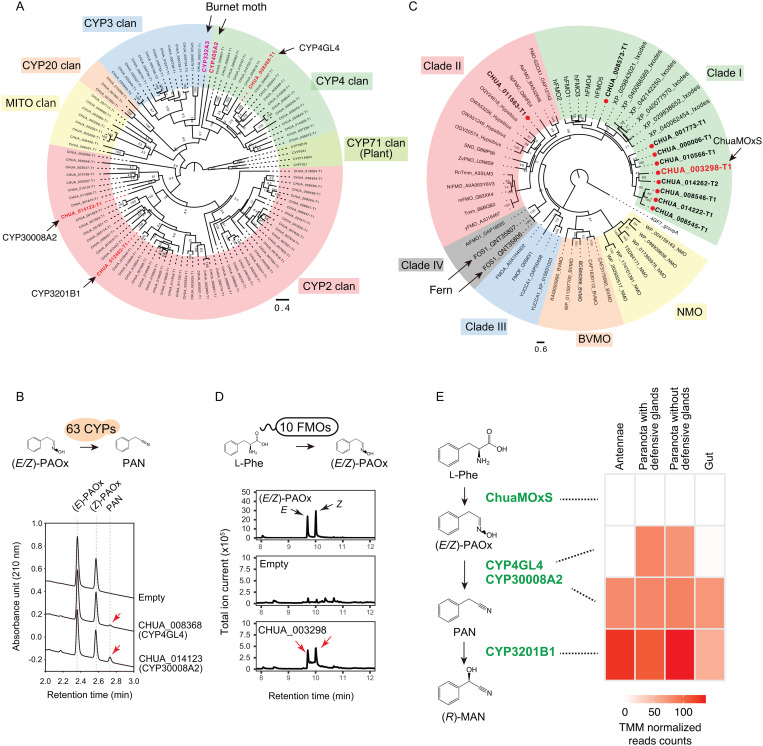
Identification of (*R*)-mandelonitrile biosynthetic enzymes from *C. hualienensis.* (A) Phylogenetic tree of cytochrome P450s (CYPs) from *C. hualienensis* and previously characterised cyanohydrin biosynthetic CYPs from the burnet moth and plants. Bar indicates 40% divergence. (B) Identification of CYPs catalysing the conversion of (*E/Z*)-PAOx into PAN. Yeast cells carrying CYPs from *C. hualienensis* expression plasmids or an empty vector were incubated with (*E/Z*)-PAOx. PAN formation was analysed using ultra-performance liquid chromatography. Reaction product peaks are indicated by red arrows. (C) Phylogenetic tree of FMOs from *C. hualienensis*, arthropods, plants, and microorganisms. Bar indicates 60% divergence. FMOs from *C. hualienensis* were labelled with red circles. (D) Identification of the (*E/Z*)-PAOx-producing FMO. *Escherichia coli* cells carrying FMOs from *C. hualienensis* expression plasmids or an empty vector were cultured. The accumulation of (*E/Z*)-PAOx was analysed using gas chromatography–mass spectrometry. Reaction product peaks are indicated by red arrows. (E) (*R*)-Mandelonitrile biosynthetic pathway in *C. hualienensis* and gene expression (TMM-normalised read counts) of biosynthetic enzyme genes.

CYPs are key biosynthetic enzymes of cyanohydrins in cyanogenic plants and burnet moths (Fig 1B) [15,40]. However, the CYPs in Myriapoda are not well understood. We searched for CYPs in millipede and centipede genomes and found 96 *C. hualienensis* genes that encode putative functional CYPs (Fig 4A). The Polydesmida millipedes *H. holstii* and *N. nodulosa* contain 119 and 80 CYPs, respectively. These numbers were greater than those of other millipedes (32–64) and centipedes (52–63) (S12A Fig). Phylogenetic analysis revealed that *C. hualienensis* CYPs (ChuaCYPs) clustered into the CYP2, CYP3, CYP4, CYP20, and mitochondrial clans (Fig 4A). CYP clans are deep gene clades observable on phylogenetic trees [41]. CYP2 clan members comprised the largest number of CYPs in *C. hualienensis* (Fig 4A). Similarly, CYP2 clan members were most abundant in other Myriapoda species (S12 Fig). However, the CYP3 and CYP4 clans are predominant in insects [42]. The difference in clan proportions in arthropods is thought to be caused by the dynamics of gene births and deaths over evolutionary time, affecting the CYPome [42].

To narrow down the CYP candidates involved in (*R*)-MAN biosynthesis, we searched for CYPs specifically expressed in paranota with defensive glands, which accumulate (*R*)-MAN, by analysing expression using RNA-seq. However, we were unable to identify such candidate CYPs. Therefore, we performed functional CYPome analysis to identify (*R*)-MAN biosynthetic CYPs. Of these 88 microsomal CYPs, 63 were cloned. To evaluate the activity of CYPs in yeast, the redox partner cytochrome P450 reductase (CPR) must be co-expressed. In *C. hualienensis*, there is one CPR gene (ChuaCPR, CHUA_001042). We generated a yeast strain, *S. cerevisiae* ChCR11, in which *ChuaCPR* was genomically introduced. The strain exhibited a higher PAN-metabolising activity than the parent strain INVSc1 when expressed *CYP3201B1* (S13 Fig), which catalyses the hydroxylation of PAN to (*R*)-MAN [23].

We evaluated the activity of 63 ChuaCYPs towards l-Phe and (*E/Z*)-PAOx using whole-cell biocatalysis, which can detect the catalytic activities of CYPs despite their production levels in yeast being below the detection limit of the CO difference-spectrum assay [43]. Although no (*E/Z*)-PAOx-producing CYPs were detected, two CYPs, CHUA_008368 and CHUA_0014123, produced PAN from (*E/Z*)-PAOx (Figs 4B and S14). CHUA_008368 and CHUA_0014123 were designated CYP4GL4 and CYP30008A2, respectively, based on the standard CYP nomenclature. CYPs were assigned to the same family and subfamily when the amino acid level sequence identity was > 40% and >55%, respectively [44].

CYP4GL4 and CYP30008A2 belong to the CYP4 and CYP2 clans, respectively. Microsomes harbouring CYP4GL4 and CYP30008A2 catalysed the formation of PAN from (*E/Z*)-PAOx (Fig 4B). CYP4GL4 and CYP30008A2 exhibited optimum pH and temperature of 8.0 and 35°C and 7.0 and 40°C, respectively (S15 Fig). The *V*_max_ and *K*_m_ towards (*E/Z*)-PAOx were as follows: CYP4GL4, *V*_max_ = 615 ± 11.6 pmol min^−1^ mg^−1^ (microsome), *K*_m_ = 8.13 ± 1.06 µM; CYP30008A2, *V*_max_ = 106 ± 4.54 pmol min^−1^ mg^−1^ (microsome), *K*_m_ = 164 ± 29.5 µM. Both P450s act on 4-hydroxyphenylacetaldoxime (4HPAOx) and indole-3-acetldoxime (IAOx) to produce the corresponding nitriles (S16 Fig), indicating that the two nitrile-synthesising CYPs act on multiple amino acid-derived aldoximes.

### Flavin-dependent monooxygenases catalysing the first (*R*)-MAN biosynthesis step

Next, we identified 10 putative functional flavin-dependent monooxygenases (FMOs) in the *C. hualienensis* genome (Fig 4D and S9 Table) as the candidates for PAOx-producing enzymes. These FMOs had the following FMO signature sequences: FAD-binding motif (GXGXXG), FMO motif (FXGXXXHXXXYK), and NADPH-binding motif (GXGXXG) (S17A Fig), and they shared 23–63% sequence identity (S17B Fig). These FMOs, except for CHUA_011563, were predicted to have a transmembrane region at their C-terminus (S9 Table) and were localised to the endoplasmic reticulum (S9 Table), similar to mammalian FMOs involved in xenobiotic metabolism [45]. However, the insect FMOs that have been characterised are soluble proteins [46–48]. FMOs are divided into six groups (A–H) based on their structures and properties [49], and group B FMOs are further divided into four clades (I–IV) [50]. Phylogenetic analyses showed that the *C. hualienensis* FMOs belonged to clade I in group B, except for CHUA_011563, which belonged to clade II in group B (Fig 4D). The other millipede and centipede genomes contained 2–14 genes encoding putative functional FMOs (S18A Fig). These FMOs clustered into clade I or II of group B, as in the case of *C. hualienensis* FMOs (S18B Fig) and were not phylogenetically related to fern FOS1 (clade IV) or microbial FMOs (Figs 4B and S18).

All *C. hualienensis* FMOs were expressed, but CHUA_003298 was expressed at trace levels and not in a tissue-specific manner (S10 Table). Therefore, all cDNAs encoding FMOs were heterologously produced in *Escherichia coli* to determine the (*E/Z*)-PAOx-producing activity of the FMOs. Among them, *E. coli* harbouring CHUA_003298 accumulated 128 ± 51 µM (*E/Z*)-PAOx in the culture (Figs 4D and S19), whereas *E. coli* harbouring the other FMOs did not (S19 Fig). Given that CHUA_003298 did not show enzymatic activity after the disruption of *E. coli* cells, biochemical characterisation of the enzyme was not performed. The accumulation of 4HPAOx and IAOx derived from l-tyrosine and l-tryptophan, respectively, was not detected (S20 Fig), indicating that CHUA_003298 has a narrow substrate specificity for producing l-Phe-derived aldoxime. We designated the PAOx-producing enzyme CHUA_003298 as “millipede aldoxime synthase (MOxS)”.

We investigated the expression of biosynthetic enzyme genes using RNA-seq (Fig 4E and S11 Table). *ChuaMOxS* was expressed at a basal level. In contrast, other enzyme genes (*CYP4GL4*, *CYP30008A2*, and *CYP3201B1*) were clearly expressed, and their expression was not restricted to paranota-containing defensive glands but also observed in other tissues.

### Distribution of MAN biosynthetic enzyme genes among Myriapod species

**Fig 5:**
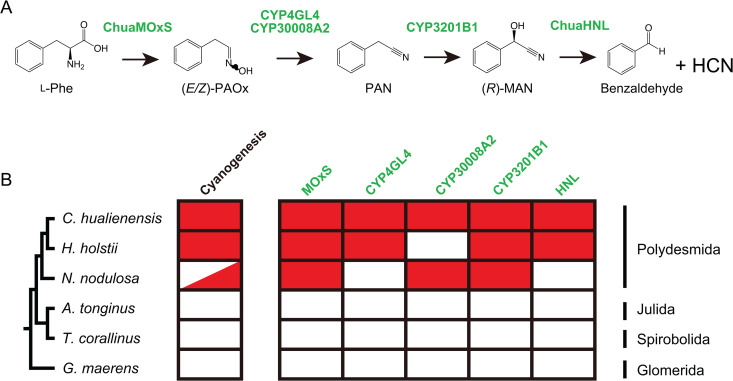
Distribution of cyanogenesis-related enzymes in millipedes. (A) Cyanogenesis pathway in *C. hualienensis*. (B) Red box indicates the presence of orthologues of *C. hualienensis* cyanogenesis-related enzyme genes.

Genes encoding cyanogenesis-related enzymes were identified in two Polydesmida millipedes, *H. holstii* and *N. nodulosa,* but not in genome-sequenced non-cyanogenic millipede species from Julida, Spirobolida, and Glomerida nor centipedes (Fig 5). ChuaMOxS (CHUA_003298) shared 82.2% and 70.6% identity towards Hho_019011 from *H. holstii* and Nno_0338811 from *N. nodulosa*, respectively (S21 Fig). *H. holstii* had CYP4GL4 (Hho_020879) and CYP30008A2 (Hho_011653) orthologues, but the CYP30008A2 orthologue lacked the N- and C-terminal region coding sequences (S22 Fig). The coding protein is likely malfunctional or the deletion may be due to gene annotation and/or sequencing errors. CYP4GL4 and Hho_020879 shared 67% identity (S23 Fig). However, *N. nodulosa* harboured the CYP30008A2 orthologue (Nno_033394) but not the CYP4GL4 orthologue. CYP30008A2 and Nno_033394 shared 72% identity (S22 Fig). Thus, the nitrile synthesis step likely differs between millipede species that utilise either one or both PAN-producing CYPs (Fig 5). *H. holstii* and *N. nodulosa* harbour an orthologue of CYP3201B1 that catalyses the conversion of PAN to (*R*)-MAN: Hho_006485 (86%) and Nno_043294 (62%), respectively. These (*R*)-MAN biosynthetic enzymes share relatively high amino acid sequence identity (>65%) among *C. hualienensis*, *H. holsti,* and *N. nodulosa* but not cyanogenesis-related enzymes from other cyanogenic plants or burnet moths. This indicates that Polydesmida millipedes independently evolved cyanogenesis-related enzyme genes.

## Discussion

*C. hualienensis* is the most extensively studied species regarding cyanogenesis among millipedes. In this study, we sequenced its genome using a combination of second-generation (Illumina) and third-generation (PacBio) sequencing technologies and obtained a high-quality and high-coverage draft genome. The *C. hualienensis* genome was the smallest (144.5 Mbp) among sequenced myriapod genomes ranging from 181 to 2530 Mbp. As myriapod genome size was clearly correlated with TE content (S3 Fig), the low TE content in the *C. hualienensis* genome likely contributed to its smallest size. The genome and biochemical characterization enabled the identification of the gene cluster containing *ChuaHNL* and its paralogous genes, as well as the PAOx- and PAN-producing enzymes, which had previously remained unclear. The discovery of cyanogenesis-related enzymes in millipedes indicated that cyanogenic millipedes evolved these enzymes independently from plants and insects.

HNL is a key enzyme that releases HCN into cyanogenic millipedes. Millipede HNLs do not exhibit sequence similarity to other proteins [17] but are conserved among cyanogenic millipedes [19]. *ChuaHNL* (CHUA_005138) and its paralogous protein-coding genes were clustered (Fig 3A). CHUA_005137 and CHUA_005136 exhibited HNL activity similar to that of ChuaHNL, and ChuaHNL and CHUA_005137 showed high *K*_m_ values toward MAN (S10A Fig). Such high *K*_m_ values are reasonable because millipede HNL acts on the high concentration of neat MAN supplied from the storage chamber and rapidly produces HCN gas and benzaldehyde, in contrast to plant-derived HNLs, which typically function at lower substrate concentrations generated by β-glucosidase–mediated hydrolysis of cyanogenic glycosides [21]. ChuaHNL was highly expressed in the paranota-containing defensive glands, whereas the expression of its paralogs was low (Fig 3B). Thus, these paralogous proteins are unlikely to accumulate in the reaction chambers of defensive glands. Although the two ChuaHNL paralogous proteins are unlikely to play important roles in millipedes, they catalyse industrially important asymmetric cyanohydrin synthesis with high enantioselectivity (S11 Fig). In particular, the kinetic parameters of CHUA_005137 were comparable with those of ChuaHNL (S10B Fig). Millipede HNLs commonly exhibit high specific activity towards (*R*)-MAN synthesis [19], whereas other cyanohydrin synthetic activities differ in terms of specific activity and stereoselectivity [18,20]. Thus, these ChuaHNL paralogous proteins may have distinct substrate specificities and could be valuable for synthesising optically pure cyanohydrins, such as pharmaceutical intermediates. Genomic analysis of cyanogenic millipedes could lead to the discovery of HNLs that are unobtainable by enzyme purification or degenerate PCR-based cloning, as described previously [17–19].

Based on their three-dimensional structure, millipede HNLs belong to the lipocalin family [18,20,21]. Lipocalins are distributed in all kingdoms of life, except for Archaea [51] and represent a large superfamily of proteins sharing a conserved scaffold; however, amino acid sequences can be highly divergent, with identities as low as 10%. Lipocalins interact with and transport small hydrophobic molecules, such as steroid hormones, odourants (e.g., pheromones), retinoids, and lipids [52]. In arthropods, some lipocalin genes are highly expressed in the antennae and pheromone glands and may function as semiochemical carrier proteins [39]. Thus, ChuaHNL paralogous proteins may interact with these small hydrophobic molecules. Several paralogues, such as CHUA_005124 and CHUA_005727, are expressed in the antennae (Fig 3B), suggesting that they function as chemosensory proteins.

Multiple lipocalin genes were found in arthropod genomes (3–49) and were clustered on some arthropod species genomes [39,53], as in the case of the HNL paralogue cluster in *C. hualienensis* and *H. holstii* (Fig 3A). The relatively large number of these genes in some arthropod species may have resulted from extensive duplication and differentiation under environmental pressure. Ancestral millipede lipocalin proteins that can bind small hydrophobic molecules may have acquired the catalytic residues necessary for HNL activity through sequential duplication of their coding genes in the genome, giving rise to highly active and stable millipede HNLs.

The pathway for cyanohydrin biosynthesis from amino acids via aldoxime and nitrile is conserved among millipedes, insects, and plants (Fig 1B). We identified a FMO, ChuaMOxS, which catalyses the formation of (*E/Z*)-PAOx (Fig 4D), the first step in the millipede cyanogenesis pathway. Animal FMOs are xenobiotic-metabolising enzymes with broad substrate specificity that play important roles in drug metabolism, particularly in the human liver [54]. Similarly, the insect FMOs characterised thus far catalyse the detoxification of insecticides and toxic plant-specialised metabolites in food [47,48,55]. In contrast to the previously characterised arthropod FMOs, ChuaMOxS is a narrow substrate-specific enzyme that produces l-Phe-derived (*E/Z*)-PAOx, as it does not produce other amino acid-derived aldoximes (S20 Fig). Aldoxime formation in cyanogenic seed plants is generally catalysed by CYPs belonging to the CYP79 family. These enzymes exhibit narrow substrate specificity, resembling that of ChuaMOxS, and specify the cyanogenic glucoside biosynthetic pathway [26,29,56]. CYP79s catalyse two successive *N*-hydroxylations of the primary amino acid and a decarboxylation step to produce aldoxime. Similarly, CYP405A2 is responsible for the formation of aldoxime in the burnet moth [31]. This is an example of convergent evolution of CYPs across the plant and animal kingdoms. However, some FMOs can produce aldoxime from amino acids, but this has rarely been reported. A group B, clade IV FMO, named FOS1, from a fern, and orchid oxime synthase (OOS), from Darwin’s orchid, catalyse the formation of aldoxime from amino acids [29,57]. The microbial FMO SCO7468 from *Streptomyces coelicolor* A3(2) catalyses the conversion of 5-dimethylallyl-l-tryptophan into the corresponding aldoxime [58]. As these FMOs are thought to catalyse two successive *N*-hydroxylations, similar to CYP79s, ChuaMOxS presumably produces (*E/Z*)-PAOx via the same reaction mechanism. These millipede, plant, and microbial FMOs are group B FMOs but are phylogenetically distant (Fig 4C), indicating that they developed through convergent evolution rather than horizontal gene transfer.

The second step in (*R*)-MAN biosynthesis is the conversion of (*E/Z*)-PAOx to PAN. We identified two novel CYPs, CYP4GL4 and CYP30008A2, which catalyse the formation of PAN from (*E/Z*)-PAOx (Fig 3B). Aldoxime dehydration is an atypical reaction for CYPs, which generally catalyse monooxygenation, but several CYPs from humans and plants catalyse aldoxime dehydration [59–63]. In the reaction, the reduction of heme iron Fe(III) to Fe(II) in the heme of CYPs enables the binding of the nitrogen atom of aldoximes to the heme iron, which allows charge transfer from Fe(II) to the aldoxime C = N bond, favouring elimination of the hydroxy group [59,64,65]. CYP4GL4 and CYP30008A2 belong to different clans and share only 20% identity at the amino acid level (Fig 3A), indicating that the two nitrile-producing enzyme genes were recruited from non-paralogous genes in millipedes. The *K*_m_ value of CYP30008A2 towards (*E/Z*)-PAOx was 164 µM, 20 times higher than the 8.13 µM of CYP4GL4 (S15 Fig). However, the *K*_m_ value of CYP30008A2 is presumably low enough to catalyse the conversion of (*E/Z*)-PAOx into PAN in millipedes because the *K*_m_ of CYP30008A2 was not high compared to the 3.9–3200 µM of plant aldoxime-metabolising CYPs [26,56,63,66,67]. The genes encoding both enzymes are similarly expressed in paranota-containing defensive glands; therefore, the contribution of each enzyme to the nitrile-synthesising step is unclear. However, both enzymes could be involved in the nitrile-producing step, considering that the other two cyanogenic millipedes, *H. holstii* and *N. nodulosa*, harboured only one of the enzyme-encoding genes.

ChuaMOxS is a narrow substrate-specificity enzyme that produces l-Phe-derived (*E/Z*)-PAOx but not aldoxime from other amino acids (S20 Fig), whereas the nitrile-producing CYP4GL4 and CYP30008A2 and the cyanohydrin-producing CYP3201B1 are broad substrate-specificity enzymes acting on multiple amino acid-derived aldoximes and nitriles (S16 Fig) [23]. Furthermore, the expression of *ChuaMOxS* was considerably lower than that of *CYP4GL4*, *CYP30008A2*, and *CYP3201B1* (Fig 4E). Given the low expression level of *ChuaMOxS* together with the high accumulation of (*R*)-MAN in the reservoir of defensive glands (Figs 1A and S4), it seems unlikely that (*R*)-MAN is synthesized in a constitutive manner. Instead, biosynthesis may be induced under specific conditions, for example, when the compound is released upon predation or during molting, where the expression of *ChuaMOxS* might be upregulated. Taken together, the narrow substrate specificity and gene expression profile of *ChuaMOxS* likely regulate the biosynthesis of (*R*)-MAN in millipedes.

Cyanogenesis-related enzyme genes were specifically found in Polydesmida but not other millipede orders (Fig 5). Furthermore, the enzyme clusters within arthropod enzymes exhibited no similarity or phylogenetic relationships with cyanogenesis-related enzymes from cyanogenic plants and insects (S12 and S18 Figs). These results indicate that Polydesmida millipedes independently evolved cyanogenesis-related enzyme genes from other cyanogenic organisms, ruling out other scenarios, such as divergent evolution or horizontal gene transfer from cyanogenic plants and the burnet moth. The independent evolution of identical enzyme activities can be either “convergent evolution” or “parallel evolution” [68]. “Parallel evolution” is used when ancestral descendants possessing distinct biochemical activities but a shared structural lineage contemporarily evolve to synthesise the same metabolite. When distinct protein structures sharing no structural similarity result in the synthesis of the same metabolite, the term “convergent evolution” is employed. Thus, millipede cyanohydrin biosynthetic enzymes are the case of both convergent and parallel evolution with plant and insect enzymes. The recruitment of FMO (MOxS) in millipedes and CYPs in higher plants and insects constitutes an example of convergent evolution, whereas MOxS and fern FOS result from parallel evolution. Cyanohydrin synthesising steps are separated into two enzymes in millipedes. Generally, higher plants and insects synthesise cyanohydrin from aldoxime by a single CYP; sugar gum (*Eucalyptus cladocalyx*) utilises CYP706C55 and CYP71B103 [69], similar to the millipede (Fig 1B). The cyanohydrin synthesising step results from parallel evolution with millipedes and sugar gum. Thus, the cyanogenesis-related enzymes emerged multiple times independently through convergent and/or parallel evolution across arthropod and plant lineages. Our results will help to understand the mechanisms underlying the evolution of metabolic pathways among arthropods and plants.

## Materials and methods

### Animals

*C. hualienensis* was collected from Kagoshima Prefecture, Japan, in November 2017 and 2018. The millipedes were reared in litter derived from the Japanese cedar *Cryptomeria japonica* D. Don at 22°C until use, as described previously [23].

### DNA preparation and genome sequencing

Genomic DNA was prepared from male millipedes for library construction. DNA samples were shipped to the Beijing Genomics Institute, Shenzhen, China, and sequenced using Illumina and PacBio Sequel sequencers. For detailed information about hybrid assembly, annotation, and other bioinformatic analysis, see S1 Text.

### Phylogenetic analysis

A comparative genome sequence analysis of *C. hualienensis* was performed using 14 genomes. Orthologous groups were identified using Orthofinder2 (v. 2.5.4) with the ‘-M msa -T raxml-ng’ flag.

### Purification of ChuaHNL and its paralogous proteins with HNL activity

*P. pastoris* transformants were inoculated into 5 mL of YPD (1% yeast extract, 2% peptone, and 2% d-glucose). After 24 h culture at 30°C with shaking at 300 rpm, cells were harvested via centrifugation at 3,000 × *g* and 4°C for 10 min, resuspended in 500 mL of BMGH in a 2-L baffled flask, and grown at 30°C with shaking at 150 rpm for 14–18 h. Then, cells were harvested via centrifugation at 3,000 × *g* and 4°C for 10 min and resuspended in 500 mL of buffered minimal methanol medium, and 1% methanol was added as an inducer every 24 h. After 5 days of culture at 28°C with shaking at 150 rpm, the culture was centrifuged at 8,000 × *g* and 4°C for 15 min. The supernatant was recovered, and the pH was adjusted to 7.5 by adding aqueous 1 M K_2_HPO_4_. Resultant insoluble materials were removed via centrifugation at 13,000 × *g* and 4°C for 30 min and directly applied to a Ni Sepharose 6 fast flow column (Cytiva, Little Chalfont, UK), which was equilibrated with 20 mM KPB, pH 7.5, containing 300 mM NaCl and 20 mM imidazole. The column was washed with the same buffer, and the absorbed proteins were eluted using a linear imidazole gradient (20–500 mM) in the same buffer. The active fractions were concentrated and desalted using an Amicon Ultra-15 centrifugal filter device with a 10,000 nominal molecular weight limit cutoff (Merck Millipore, Billerica, MA, USA). It was loaded onto a Mono Q 5/50 column (Cytiva) equilibrated with 20 mM tricine-NaOH (pH 8.5) and eluted using a linear gradient of NaCl (0–500 mM) in the same buffer. The active fractions were pooled and concentrated using a centrifugal filtration device (Amicon Ultra 0.5 Centrifugal Filter Unit with ultracel-10 membrane; Merck Millipore).

### HNL assay

Enzyme activities for (*R*)-MAN synthesis and MAN cleavage were assayed using high-performance liquid chromatography and spectrophotometry, respectively. HNL activity during (*R*)-MAN synthesis was measured as previously reported [21], with slight modifications. In brief, the reaction (total volume = 0.25 mL) was initiated by adding potassium cyanide (100 mM) in citrate buffer (400 mM, pH 4.0) containing benzaldehyde (50 mM; 12.5 μL of 1 M benzaldehyde dissolved in dimethyl sulfoxide), and enzyme solution (0.1–1.0 U/mL). The final dimethyl sulfoxide concentration in the mixture was 5% (v/v). The mixture was incubated at 25°C for 5 min, and then aliquots (50 μL) of reactant were mixed with a nine-fold volume (450 µL) of a *n*-hexane:2-propanol (85:15) mixture containing 5 mM *p*-xylyl cyanide as an internal standard.

Finally, the organic phase was analysed using a high-performance liquid chromatography instrument (UFLC Prominence Liquid Chromatograph LC-20AD, Shimadzu, Kyoto, Japan) equipped with a chiral column (CHIRALCEL OJ-H, Daicel, Osaka, Japan; 250 mm length × 4.6 mm inner diameter (i.d.), particle size 5 µm). The amount of (*R*)-MAN was estimated using a standard curve obtained from the peak areas and concentration ratios of the authentic compounds and the internal standard. One unit of synthesis activity was defined as the amount of the enzyme that synthesises 1 μmol of (*R*)-MAN from benzaldehyde and potassium cyanide per minute.

For the MAN cleavage activity assay, the reaction was initiated by adding the enzyme sample to a reaction mixture (1 mL) consisting of (*R,S*)-MAN (10 mM) in citrate buffer (100 mM, pH 5.0). The reaction mixture contained 5% (v/v) dimethyl sulfoxide at the final concentration. The reaction velocity of benzaldehyde formation was monitored using an Evolution 201 ultraviolet-visible spectrophotometer (Thermo Fisher Scientific) at 25°C and 280 nm for 1 min (extinction coefficient of benzaldehyde = 1,352 L mol^−1^ cm^−1^) [21]. One unit of cleavage activity was defined as the amount of enzyme that produces 1 μmol of benzaldehyde from (*R,S*)-MAN per minute.

### Collection of ChuaCYP sequences from myriapod genomes

CYPs from *C. hualienensis* and genome-sequenced myriapods were searched via BLASTP using CYP3201B1 (CYP2 clan, BAV93938.1), CYP3193A1 (CYP3 clan, BAV93917.1), CYP4GQ1 (CYP4 clan, BAV93949.1), CYP20A1, (CYP20 clan, BAV93936.1), and CYP302A1 (mitochondrial clan, BAV93914.1) from *C. hualienensis*. The *E*-value threshold was 1.0 × 10^−10^. CYPs with sequences that were too short (<400 aa) or too long (>600 aa) were eliminated from the dataset as putative malfunctioning proteins because animal P450s are 500 amino acids in length [70]. CYPs from *S. martima* were derived from a previous study [42].

### Heterologous production of ChuaCYPs in *S. cerevisiae* and identification of aldoxime- and nitrile-producing CYPs

Expression vectors were constructed to co-express cDNAs encoding ChuaCPR and ChuaCYPs. cDNAs encoding ChuaCPR (CHUA_001042) and ChuaCYPs were amplified via PCR and cloned into the pYeDP60 vector [71] using the NEBuilder HiFi DNA Assembly Master Mix (New England Biolabs, Ipswich, MA, USA). The inserted DNA sequences were confirmed via Sanger sequencing. ChuaCPR with the GAL10-CYC1 promoter and PGK terminator region was amplified via PCR, and the amplicon was cloned into the SphI site of pAUR101 (Takara, Shiga, Japan) to generate pAUR-ChuaCPR. The plasmid was linearised using StuI and integrated into the genome DNA of *S. cerevisiae* INVScI (Thermo Fisher Scientific) through homologous recombination. The transformants were selected on a YPD-agar plate containing 0.4 µg/mL of aureobasidin A at 30°C for 3 days. Genome integration of ChuaCPR was confirmed through PCR, and the selected strain was named ChCR11 for subsequent experiments.

pYeDP60 vectors carrying cDNAs encoding CYPs were used to transform ChCR11. The transformants were selected on SGI-agar plates (0.67% yeast nitrogen base without amino acids, 0.1% casamino acid, 40 µg/mL l-tryptophan, 2% d-glucose, and 1.5% agar) at 30°C for 2 days. Transformants were inoculated into 0.5 mL of SGI medium (0.67% yeast nitrogen base without amino acids, 0.1% casamino acid, 40 µg/mL l-tryptophan, and 2% d-glucose) and cultured at 30°C for 24 h. Cells were harvested from 0.1 mL culture and resuspended in 0.5 mL of 2 × SLI medium (1.34% (w/v) yeast nitrogen base without amino acids, 0.2% (w/v) casamino acid, 80 µg/mL l-tryptophan, and 4% (w/v) galactose) supplemented with 0.2% (w/v) raffinose. After culturing at 30°C for 24 h, the cells were harvested and resuspended in 0.2 mL of 2 mM l-Phe, 1 mM (*E/Z*)-PAOx, or 1 mM PAN in 50 mM KPB (pH 7.0) containing 20 mM d-glucose. The reaction was performed at 30°C with shaking at 1,200 rpm for 180 min.

The production of (*E/Z*)-PAOx from l-Phe was analysed using liquid chromatography–mass spectrometry (LC–MS) (Nexera HPLC coupled with an LCMS-2020, Shimadzu), equipped with a COSMOSIL 3C18-MS-II packed column (100 mm × 2.0 mm i.d., particle size 3 µm; Nacalai Tesque, Kyoto, Japan). The separation conditions were as follows: column oven temperature, 40°C; mobile phase A, 0.1% formic acid in water; mobile phase B, acetonitrile; 10–60% linear gradient of B for 7.5 min and 98% B for 2.5 min, delivered at 0.4 mL/min. MS was simultaneously performed in the negative-ion mode using an LCMS-2020 apparatus (Shimadzu) via electrospray ionisation. (*E/Z*)-PAOx ionised in the positive-ion mode was monitored in the extracted ion *m/z* 136 [M + H]^+^. The production of PAN from (*E/Z*)-PAOx and MAN from PAN was analysed using an ACQUITY UPLC H-Class system (Waters, Milford, MA, USA) equipped with a COSMOCORE 2.6 C_18_ column (50 mm × 2.1 mm i.d., particle size 2.6 µm; Nacalai Tesque) under the following conditions: column oven temperature, 40°C; mobile phase A, 0.1% formic acid in water; mobile phase B, acetonitrile; 10–60% linear gradient of B for 4 min and 60% B for 0.5 min, delivered at 0.4 mL/min. Aldoximes and nitriles were quantified at 210 nm.

### Preparation of microsomes harbouring CYP4GL4 and CYP30008A2 from yeast

Microsomes harbouring CYP4GL4 (CHUA_008638) and CYP30008A2 (CHUA_014123) were prepared from *S. cerevisiae* ChCR11, following a previously described method [63]. The yeast cells were cultured in 2 mL SGI medium at 30°C for 24 h. The culture was transferred to 50 mL SGI medium at 30°C for 16 h. Yeast cells were harvested via centrifugation at 5,000 × *g* and 4°C for 10 min and resuspended in 500 mL of 2 × SLI medium supplemented with 0.2% (w/v) raffinose in a 2-L baffled flask at 30°C for 24 h. Yeast cells were harvested via centrifugation (5,000 × *g*, 10 min, 4°C), washed with 50 mM HEPES-NaOH (pH 7.6) containing 20% glycerol, and weighed to obtain the wet cell weight. The cells were then resuspended in 1 mL of 50 mM HEPES-NaOH (pH 7.6) containing 20% glycerol, 1 mM DTT, and an appropriate amount of proteinase inhibitor cocktail (complete Mini EDTA-Free; Merck KGaA, Darmstadt, Germany) per gram of wet cells. The cells were disrupted using a Multi-beads Shocker system (Yasui Kikai, Osaka, Japan) and glass beads (diameter 0.5 mm), as described previously [18]. The cell debris and beads were precipitated via centrifugation (10,000 × *g*, 10 min, 4°C), and the supernatant was ultracentrifuged (150,000 × *g*, 60 min, 4°C) to precipitate the fraction of intact microsomes. The microsomes were resuspended in 50 mM HEPES-NaOH (pH 7.6), containing 20% glycerol and 1 mM DTT and stored at −80°C until further use. The protein concentration of the microsomes was determined using the TaKaRa Bradford Protein Assay Kit (Takara) with bovine serum albumin as the standard.

### Aldoxime dehydratase assay

CYP4GL4 and CYP30008A2 activity was evaluated at optimal pH and temperature. A 100 µL reaction mixture containing the microsomal fraction harbouring CYP4GL4 (5 mg/mL), 50 mM KPB (pH 8.0), 1 mM NADPH, and 1 mM aldoxime was incubated at 35°C for 30 min. After the pre-incubation at 35°C for 5 min, the reaction was started by adding the microsomes. A 100 µL reaction mixture containing the microsomal fraction harbouring CYP30008A2 (5 mg/mL), 50 mM KPB (pH 7.0), 1 mM NADPH, and 1 mM aldoxime was incubated at 40°C for 30 min. After pre-incubation at 40°C for 5 min, the reaction was initiated by adding microsomes. The reaction was terminated by adding 0.1 mL of 0.2% formic acid in 40% acetonitrile. The resulting insoluble materials were precipitated via centrifugation (21,500 × *g*, 10 min, 4°C). The supernatant was analysed using an ACQUITY UPLC H-Class system (Waters) equipped with a COSMOCORE 2.6 C_18_ column (50 mm × 2.1 mm i.d., particle size 2.6 µm; Nacalai Tesque) under the following conditions: column oven temperature, 40°C; mobile phase A, 0.1% formic acid in water; mobile phase B, acetonitrile; 10–60% linear gradient of B for 4 min and 60% B for 0.5 min, delivered at 0.4 mL/min. Aldoximes and nitriles were quantified at 210 nm. The nitriles were quantified using standard curves generated from authentic compounds (PAN, Tokyo Chemical Industry, Tokyo, Japan; 4-hydroxyphenylacetonitrile, Tokyo Chemical Industry; indole-3-acetonitrile, Sigma-Aldrich, St Louis, MO, USA). To determine *K*_m_ and *k*_cat_, the reactions were conducted under standard assay conditions using 10–2000 µM (*E/Z*)-PAOx. Aldoximes were chemically synthesised as previously described [63]. *K*_m_ and *k*_cat_ were determined by curve fitting the data using the drc package (version 3.0-1) [72] in R (4.1.2) [73] and the Michaelis–Menten equation as previously described [74].

### Collection of FMO sequences from myriapod genomes

FMOs from *C. hualienensis* and genome-sequenced myriapods were searched using BLASTP using FMO1 from *Homo sapiens* (group B clade I, UPI0003EAECD6), SNO from *Tyria jacobaeae* (group B, clade II, Q8MP06), FOS1 from *Phlebodium aureum* (group B, clade IV, QNT35807), and SCO7468 from *S. coelicolor* (Q8CJJ9). The *E*-value threshold was 1.0 × 10^−10^. The FMOs with short (<350 aa) and long (>550 aa) sequences were eliminated from the dataset because group B FMOs were 420–520 amino acids in length [50]. The transmembrane region and subcellular localisation were predicted using Phobius [75] and DeepLoc 2.1 [76], respectively.

### Identification of ChuaFMOs catalysing (*E/Z*)-PAOx formation in *E. coli*

Each *E. coli* BL21(DE3) transformant carrying pGro7 (Takara) and ChuaFMO expression plasmid was inoculated into LB containing 1% (w/v) glucose, kanamycin (50 µg/mL), and chloramphenicol (34 µg/mL) and cultured overnight at 37°C. Ten microlitres of culture was transferred to 2 mL of a TB-based autoinduction medium containing 2 mg/mL l-arabinose and cultured at 37°C for 2 h and further cultured at 16°C for 22 h. The culture was centrifuged at 5,000 × *g* and 4°C for 15 min, and the supernatant was extracted twice with 1 mL of ethyl acetate. The organic layer was collected and evaporated under a nitrogen atmosphere. The samples were dissolved in 100 µL of methanol, and a portion of the solution (1 µL) was analysed using an Agilent 7890A GC equipped with an HP-5ms column (30 m × 0.25 mm i.d.; 0.25 μm film thickness, Agilent Technologies). The column oven was programmed from 60°C for 2 min, then at 10°C min^−1^ to 290°C, and maintained for 5 min. Helium was used as the carrier gas at a flow rate of 1.0 ml min^−1^. MS was performed simultaneously using a GC system coupled with an Agilent 5975C inert XL EI/CI MSD equipped with a triple-axis detector (Agilent Technologies). All mass spectra were acquired in electron impact mode (ionisation energy: 70 eV). (*E/Z*)-PAOx was identified from the mass spectra, retention times, and comparisons with authentic compounds.

### Substrate specificity of ChuaMOxS (CHUA_003298)

*E. coli* BL21(DE3) carrying pGro7 (Takara) and p28-ChuaMOxS was inoculated into LB containing 1% (w/v) d-glucose, kanamycin (50 µg/mL), and chloramphenicol (34 µg/mL) and was cultured overnight at 37°C. Twenty microlitres of the culture was transferred to 2 mL of a TB-based autoinduction medium containing 2 mg/mL l-arabinose and cultured at 37°C for 2 h and further cultured at 16°C for 22 h. A portion (500 µL) of culture was mixed with an equal volume of 40% acetonitrile containing 0.2% formic acid and centrifuged at 21,400 × *g* and 4°C for 15 min. The supernatant was collected, and the presence of PAOx, 4-hydroxyPAOx, or IAOx in the medium was detected using an LC–MS system equipped with a COSMOSIL 3C18-EB packed column (100 × 2.0 mm i.d., particle size 3 µm; Nacalai Tesuque). The separation conditions were as follows: column oven temperature, 40°C; mobile phase A, 0.1% formic acid in water; mobile phase B, acetonitrile; 20–98% linear gradient of B for 7.5 min and 98% B for 2.5 min, delivered at 0.4 mL/min. MS was simultaneously performed in positive-ion mode, using an LCMS-2020 apparatus (Shimadzu), via electrospray ionisation (interface voltage, 3 kV; interface temperature, 300°C; DL temperature, 250°C; heat block temperature, 400°C; nebulising gas, 3 L/min; drying gas, 10 L/min; heating gas, 10 L/min). Aldoximes ionised in positive-ion mode were monitored in the extracted ions: PAOx, *m/z* 136 [M + H]^+^; 4HPAOx, *m/z* 152 [M + H]^+^; and IAOx, *m/z* 175 [M + H]^+^. The (*E/Z*)-PAOx accumulated in the medium was quantified using standard curves generated from authentic compounds.

### Phylogenetic analysis

Phylogenetic analyses of HNL, P450s, and FMOs were performed. Multiple sequence alignments were performed using MAFFT software [77]. A phylogenetic tree was constructed using the maximum-likelihood method and RAxML-NG [78] with the best-fit amino acid substitution model (HNL; WAG + I + G4 + F, P450; LG + I + G4 + F, FMO; LG + G4 + F) determined based on ModelTest-NG [79] and the Akaike Information Criterion. The tree was evaluated using bootstrap analysis with 500 (P450) or 1000 (HNL and FMO) replicates.

### Statistical analysis

Statistical analysis was performed using the multcomp package [80] in R (version 4.12), and differences were analysed using Welch’s t-test or Tukey’s honest significant difference test. Differences were considered statistically significant at P < 0.05.

## Supporting information

S1 Text Additional methods used in this study. (DOCX)

S1 Fig Overview of the processing pipeline used for the assembly of the *Chamberlinius hualienensis genome* (see materials and methods for detail). (TIF)

S2 Fig GenomeScope plot of the 21-mer content within the *C. hualienensis* genome. Dataset show the fit of the GenomeScope model (black) based on 21-kmers in Illumina HiSeq sequence reads.
(TIF)

S3 Fig Correlations of genome sizes and TE contents among millipedes and centipedes. (TIF)

S4 Fig Body weight and accumulation of (*R*)-mandelonitrile in *Chamberlinius hualienensis.* Each boxplot shows median (the center horizontal), interquartile range (upper and lower edges of the box), and 1.5 times the interquartile range (whisker) (n = 6). Welch’s *t* test was used to analyze the data for significant differences between male and female. *P* values less than 0.05 were considered statistically significant.
(TIF)

S5 Fig Amino acid sequence identity matrix of ChuaHNL and its paralogous proteins. (TIF)

S6 Fig Amino acid sequence alignment of ChuaHNL and its paralogous proteins. A red background identifies strictly conserved residues; red letters indicate residues well conserved within a group, according to a Raisler matrix; and all other amino acids are shown in black. Residues conserved between groups are boxed with a blue line. The secondary structure elements of ChuaHNL (CHUA_005138; PDB ID: 6KQW) are shown as follows: α-helices, medium squiggles with α symbols; 3_10_-helices, squiggles with η symbols; β-strands, arrows with β symbols; strict β-turns, TT letters. Identical residues are highlighted with red boxes and white text. Stars on the sequence indicated the catalytic residues of ChuaHNL.
(TIF)

S7 Fig Phylogenetic tree of millipede HNLs, their paralogous proteins, and lipocalins from animals (arthropoda and chordata), plants, and bacteria. The bar indicates 50% divergence.
(TIF)

S8 Fig Three dimensional structures of hydroxynitrile lyase (HNL) from millipede, plant, and microorganism. (TIF)

S9 Fig Heterologous production of ChuaHNL and its paralogous proteins in *Pichia pastoris.* (TIF)

S10 Fig Substrate saturation curves for CHUA_005138 (ChuaHNL), CHUA_005137, and CHUA_005136 with (*R,S*)-mandelonitrile (mandelonitrile cleavage reaction) (A) or benzaldehyde ((*R*)-mandelonitrile synthetic reaction) (B). (TIF)

S11 Fig (*R*)-mandelonitrile synthetic reaction catalyzed by CHUA_005138 (ChuaHNL), CHUA_005137, and CHUA_005136. The reaction products were extracted and analyzed by a high performance liquid chromatograph equipped with a chiral column.
(TIF)

S12 Fig Phylogenetic tree of cytochrome P450s. (A) Number of cytochrome P450s from Myriapoda species (*C. hualienensis*, *H. holstii*, *N. nodulosa*, *A. tonginus*, *T. corallinus*, *G. maerens*, *L. niger*, *R. immarginata*, *S. martima*, *T. tuberculata*). (B) Phylogenetic tree of cytochrome P450s from myriapods, insects, plants, and microorganisms. The bar indicates 70% divergence.
(TIF)

S13 Fig Comparison of phenylacetonitrile (PAN) metabolizing activity of *Saccharomyces cerevisiae* ChCR11 and INVSc1 expressing *CYP3201B1.* Yeast cells carrying the CYP3201B1 expression plasmid or an empty vector were incubated with PAN. PAN after the reaction was quantified using ultra-performance liquid chromatography. Reactions were carried out in triplicate (*n* = 3), error bars show the standard deviation of the replicate measurements, the error bar centers are the means of the replicate measurements, and the replicate measurements are represented as black dots. Bars labelled with different letters indicate a significant difference (*P* < 0.05) as determined by Tukey’s honest significant difference test.
(TIF)

S14 Fig Formation of phenylacetonitrile (PAN) from (*E/Z*)-phenylacetaldoxime by yeast harboring CHUA_008368 (CYP4GL4) and CHUA_014123 (CYP30008A2). Yeast cells carrying the CHUA_008368 and CHUA_014123 expression plasmid or an empty vector were incubated with (*E/Z*)-phenylacetaldoxime. The formation of PAN was quantified using ultra-performance liquid chromatography. Reactions were carried out in triplicate (*n* = 3), error bars show the standard deviation of the replicate measurements, the error bar centers are the means of the replicate measurements, and the replicate measurements are represented as black dots. nd, not detected.
(TIF)

S15 Fig Characterization of CYP4GL4 (CHUA_008368) and CYP30008A2 (CHUA_014123) catalyzing dehydration of (*E/Z*)-phenylacetaldoxime (PAOx) into phenylacetonitrile. Reactions were carried out in triplicate (*n* = 3), error bars show the standard deviation of the replicate measurements, and the error bar centers are the means of the replicate measurements.
(TIF)

S16 Fig Substrate specificity of CYP4GL4 (CHUA_008368) and CYP30008A2 (CHUA_014123) toward aromatic aldoximes. A. Microsome harboring CYP4GL4 and CYP3008A2 were incubated with 1 mM aldoximes in the presence of NADPH. Corresponding nitriles produced from (*E/Z*)-phenylacetaldoxime (PAOx), (*E/Z*)-4-hydroxyphenylacetaldoxime (4HPAOx), and (*E/Z*)-indole-3-acetaldoxime (IAOx) were quantified using ultra performance liquid chromatography. The activity toward PAOx was defined as 100%. Reactions were carried out in triplicate (*n* = 3), error bars show the standard deviation of the replicate measurements, the error bar centers are the means of the replicate measurements, and the replicate measurements are represented as black dots. B. The reactions catalyzed by the two enzymes.
(TIF)

S17 Fig Amino acid sequence alignment (A) and sequence identity matrix (B) of *Chamberliniensis hualienensis* flavin-dependent monooxygenases. A red background identifies strictly conserved residues; red letters indicate residues well conserved within a group, according to a Raisler matrix; and all other amino acids are shown in black. Residues conserved between groups are boxed with a blue line.
(TIF)

S18 Fig Flavin-dependent monooxygenases in myriapods and other species. (A) Number of flavin-dependent monooxygenases from Myriapoda species (*C. hualienensis*, *H. holstii*, *N. nodulosa*, *A. tonginus*, *T. corallinus*, *G. maerens*, *L. niger*, *R. immarginata*, *S. martima*, *T. tuberculata*). (B) Phylogenetic tree of flavin-dependent monooxygenases from myriapods and other animals, plants, and microorganisms. The bar indicates 70% divergence.
(TIF)

S19 Fig Identification of phenylacetaldoxime (PAOx)-producing flavin dependent monooxygenase from *Chamberlinius hualienensis.* (A) *Escherichia coli* cells carrying FMOs from *C. hualienensis* expression plasmids or an empty vector were cultured. The accumulation of (*E/Z*)-PAOx was analysed using gas chromatography–mass spectrometry. Reaction product peaks are indicated by red arrows. (B) The mass spectra of authentic PAOx and reaction products.
(TIF)

S20 Fig Detection of aromatic amino acids-derived aldoximes after the culture of *Escherichia coli* harboring millipede aldoxime synthase (MOxS, CHUA_003298). Accumulation of (*E/Z*)-phenylacetaldoxime, (*E/Z*)-4-hydroxyphenylacetaldoxime, and (*E/Z*)-indole-3-acetaldoxime in the medium after the culture of *E. coli* BL21(DE3) carrying pGro7 and empty pET28 plasmid or pET28 carrying CHUA_003298. Selected ion monitoring was used to detect (*E/Z*)-phenylacetaldoxime with *m/z* 136 [M + H]^+^, (*E/Z*)-4-hydroxyphenylacetaldoxime with *m/z* 152 [M + H]^+^, and (*E/Z*)-indole-3-acetaldoxime with *m/z* 175 [M + H]^+^. Aldoximes detected were indicated by red arrows.
(TIF)

S21 Fig Amino acid sequence alignment (A) and amino acid sequence identity (B) of CHUA_003298 (ChuaMOxS) from *Chamberlinius hualienensis*, Hho_019011 from *Helicorthomorpha holstii*, and Nno_033811 from *Niponia nodulosa.* (TIF)

S22 Fig Amino acid sequence alignment (A) and and amino acid sequence identity (B) of CHUA_014123 (CYP30008A2) from *Chamberlinius hualienensis* and its orthorogous proteins, Hho_011653 from *Helicorthomorpha holstii* and Nno_033394 from *Niponia nodulosa.* (TIF)

S23 Fig Amino acid sequence alignment (A) and amino acid sequence identity (B) of CHUA_008368 (CYP4GL4) from *Chamberlinius hualienensis*, Hho_020879 from *Helicorthomorpha holstii.* (TIF)

S1 Table Shotgun sequencing summary statistics (Illumina). (XLSX)

S2 Table Shotgun sequencing summary statistics (PacBio). (XLSX)

S3 Table TE contents of millipede and centipede. (XLSX)

S4 Table RNA-seq for gene annotation. (XLSX)

S5 Table Purification of N-terminal His-tagged ChuaHNL (CHUA_005138) from 1.5 L of *Pichia pastoris* culture supernatant. (XLSX)

S6 Table Purification of N-terminal His-tagged CHUA_005137 from 1.5 L of *Pichia pastoris* culture supernatant. (XLSX)

S7 Table Purification of N-terminal His-tagged CHUA_005136 from 1.5 L of *Pichia pastoris* culture supernatant. (XLSX)

S8 Table RNA-seq for gene expression analysis. (XLSX)

S9 Table Characteristics of flavin-dependent monooxygenases (FMOs) from *Chamberlinius hualienensis.* (XLSX)

S10 Table TMM normalized read counts of flavin-dependent monooxygenases (FMOs). (XLSX)

S11 Table TMM normalized read counts of (*R*)-mandelonitrile biosynthetic genes. (XLSX)

S1 Data Numerical data underlying Figs 3, 4E, S4, S8, S10, S12, S13, S14, and S16. (XLSX)
