# Supplementary material for: Cyanogenic millipede genome illuminates convergent evolution of cyanogenesis-related enzymes
Source: PLoS Genet. 2025 Nov 24;21(11):e1011955. doi: 10.1371/journal.pgen.1011955 (PMC12671775; doi:10.1371/journal.pgen.1011955)
Supplement: S1 Text — (DOCX) [file pgen.1011955.s001.docx]

**S1 text**

**Chemicals**

(*E/Z*)-PAOx and (*E/Z*)-4-hydroxyphenylacetaldoxime (4HPAOx) were synthesized by condensation of hydroxylamine with phenylacetaldehyde and 4-hydroxyphenylacetaldehyde, respectively [1]. 4-Hydroxyphenylacetaldehyde was prepared from L-tyrosine via sodium hypochlorite oxidation [2]. (*E/Z*)-Indole-3-acetaldoxime (IAOx) was synthesized by condensation of hydroxylamine with the corresponding aldehyde derived from 2-(1*H*-indol-3-yl)ethanol [3]. Other chemicals were purchased from commercial suppliers.

**DNA preparation and genome sequencing**

Genomic DNA was prepared from male millipedes for library construction. To avoid microbial contamination from the digestive system, the animals were degutted in phosphate-buffered saline before being immediately frozen in liquid nitrogen for storage at −80°C until use. Degutted millipedes were ground in liquid nitrogen using a mortar and pestle. Genomic DNA was purified using the Blood & Cell Culture DNA Maxi Kit (Qiagen, Valencia, CA, USA). DNA samples were shipped to the Beijing Genomics Institute, Shenzhen, China, and sequenced using Illumina and PacBio Sequel sequencers.

**Genome size estimation by *k*-mer analysis**

To estimate the genome size of *C. hualienensis*, *k*-mers in sequencing reads (Illumina short reads, 270 bp library) were counted using jellyfish 2.2.10 [4]. A frequency histogram of the 21-mers was obtained and analysed using GenomeScope 1.0 [5].

**Genome assembly**

First, mitochondrial genome-derived reads were removed from the raw read sequences. Illumina and PacBio reads were mapped to the assembled mitochondrial genome using BWA-MEM 0.7.17 [6] and Mimimap2.1, respectively [7]. The mitochondrial genome-filtered Illumina paired-end reads were assembled using Platanus 1.2.4 [8]. The resultant contigs and PacBio Sequel reads longer than 1 kb were applied to the hybrid assembly using the DBG2OLC and Sparc software pipelines [9]. The PacBio Sequel reads were mapped to contigs using minimap and polished twice using Racon. Next, 300 × paired-end reads were mapped to contigs using BWA-MEM, and the contigs were polished twice using Pilon [10]. Finally, 33 × mate-paired reads were mapped to polished contigs, which were scaffolded using BESST [11]. Scaffold completeness was assessed using the BUSCO [12] and Arthropoda dataset odb9 [13].

**RNA preparation and RNA sequencing**

Total RNA was prepared from antennae from male and female, segments with or without defensive glands, and gut using the RNeasy mini Kit (Qiagen). Purified total RNA was sequenced at the Beijing Genomics Institute using a Novaseq 6000 (Illumina, Hayward, CA, USA).

**RNA-seq assembly**

RNA-seq data of *C. hualienensis* (S4 Table) were assembled using the Trinity-V2.8.4 [14] with --jaccard_clip option.

**Annotation of repetitive sequences**

De novo and homology-based approaches were integrated to search for transposable elements and annotate repetitive sequences. RepeatModeler version 2.0.1 [15] was run on the unannotated assembly to identify and classify de novo repeat families. RepeatModeler employs two de novo repeat-finding programs, RECON version 1.08 and RepeatScout version 1.0.5, to identify repeat element boundaries and build consensus models of putative interspersed repeats. The sequence was aligned to GenBank’s non-redundant protein database using diamond BLASTX with an E-value cutoff of 1 × 10^−5^ (--sensitive) to ensure that repeat sequences in the library did not contain large families of protein-coding genes that are not transposable elements. Repeat masking was performed on the assembled genome using RepeatMasker version 4.0.9 (http://www.repeatmasker.org/) against repetitive sequences in the RepeatMasker consensus library (20150807; www.girinst.org) and a custom species-specific repeat library generated using RepeatModeler.

**Gene prediction and annotation**

To predict the genes, we employed the funannotate pipeline (https://github.com/nextgenusfs/funannotate). The pipeline utilises several ab initio gene predictors, including Augustus, GeneMark-ET v4.46, snap, glimmerHMM, and the PASA annotation pipeline. Augustus and GeneMark-ET were pretrained using BRAKER software. RNA-seq reads (Table S4) were mapped to scaffolds using STAR v2.7.7a. Resultant BAM files were merged and applied to BRAKER software. De novo RNA-seq assembly of the millipede was applied to the funannotate package.

**Extraction and quantification of (*R*)-MAN from *C. hualienensis***

The millipedes were separated into male and female. Their body weight was weighed, and MAN was extracted with 200 µL of MeOH containing 1 mM PAN per 100 mg body weight. A portion (2 µL) of the extract was analyzed using an Nexera UPLC (Shimadzu, Kyoto, Japan) system equipped with a COSMOCORE 2.6C_18_ column (50 mm × 2.1 mm i.d., particle size 2.6 µm; Nacalai Tesque, Kyoto, Japan) under the following conditions: column oven temperature of 40 °C; mobile phase A, 0.1% formic acid in water, mobile phase B, acetonitrile, and 10–60% linear gradient of B for 4 min and 60% B for 0.5 min delivered at 0.4 mL/min. The amount of (*R*)-MAN was estimated using the standard curve obtained from the peak area ratio and concentration ratio of the authentic compounds and the internal standard.

**Heterologous production of ChuaHNL and its paralogous proteins in *P. pastoris***

ChuaHNL and its paralogous proteins were heterologously produced as N-terminal His-tagged proteins as previously described [16]. Signal peptides were predicted using the SignalP 6.0 server (https://services.healthtech.dtu.dk/services/SignalP-6.0/). Next, ChuaHNL paralogous protein-coding sequences without signal peptides were synthesised, and the codons were optimised for expression in *P. pastoris* using GeneArt Strings (Thermo Fisher Scientific, Waltham, MA, USA). pPICZαA was linearised via inverse PCR using pPICZα-His-Syn-ChuaHNL as a template DNA (Zhai et al., 2019). The synthesised DNA fragments were cloned into a linearised vector using an In-Fusion HD Cloning Kit (Clontech Laboratories, Palo Alto, CA, USA) or the NEBuilder HiFi DNA Assembly Master Mix. The inserted DNA sequence was verified using Sanger sequencing.

The constructed vectors were linearised via digestion with SacI and transformed into *P. pastoris* PpPDI/GS115 cells [16] harbouring genomic DNA-integrated *AOX1::PpPDI*, using a Pichia EasyComp Transformation Kit (Thermo Fisher Scientiﬁc), according to the manufacturer’s instructions. Transformants were selected on YPDS (1% yeast extract, 2% peptone, 2% D-glucose, and 1 M sorbitol) agar medium containing 100 µg/mL zeocine. His-tagged recombinant protein production was evaluated as follows. Selected *P. pastoris* transformants were inoculated into 2 mL of YPD and cultured at 30°C for 16 h. Then, 100 mL of buffered minimal glycerol medium [BMGH; 100 mM potassium phosphate buffer (KPB; pH 7.0), 1.34% yeast nitrogen base without amino acid, 4×10^−5^% biotin, 0.004% L-histidine, and 1.0% glycerol] in a 500 mL baffled flask.

The cells were harvested via centrifugation and resuspended in buffered minimal methanol medium (the same as BMGH, but 1% (v/v) methanol was added instead of 1% (v/v) glycerol) at OD_600_ = 5). After 6 days culture at 28°C with shaking at 150 rpm, the culture was centrifuged at 8,000 × *g* and 4°C for 15 min. The supernatant was recovered, and the pH was adjusted to 7.2–7.5 by adding 1 M K_2_HPO_4_. Resultant insoluble materials were removed via centrifugation at 20,000 × *g* and 4°C for 15 min. To concentrate the His-tagged proteins secreted into the medium, the supernatant was applied to a column packed with 1 mL complete His-Tag Purification Resin (Roche Applied Science, Basel, Switzerland), which was equilibrated with 20 mM KPB (pH 7.5) containing 300 mM NaCl and 20 mM imidazole. The column was washed with the same buffer, and the absorbed proteins were eluted with 5 mL of the same buffer containing 300 mM imidazole.

**S1 Text References**

1. Asano Y, Kato Y. *Z*-Phenylacetaldoxime degradation by a novel aldoxime dehydratase from *Bacillus* sp. strain OxB-1. FEMS Microbiol Lett. 1998;158: 185–190. doi:10.1016/s0378-1097(97)00520-x

2. Maresh JJ, Crowe SO, Ralko AA, Aparece MD, Murphy CM, Krzeszowiec M, et al. Facile one-pot synthesis of tetrahydroisoquinolines from amino acids via hypochlorite-mediated decarboxylation and Pictet–Spengler condensation. Tetrahedron Lett. 2014;55: 5047–5051. doi:10.1016/j.tetlet.2014.07.043

3. Sugawara S, Hishiyama S, Jikumaru Y, Hanada A, Nishimura T, Koshiba T, et al. Biochemical analyses of indole-3-acetaldoxime-dependent auxin biosynthesis in *Arabidopsis*. Proc National Acad Sci. 2009;106: 5430–5435. doi:10.1073/pnas.0811226106

4. Marçais G, Kingsford C. A fast, lock-free approach for efficient parallel counting of occurrences of k-mers. Bioinformatics. 2011;27: 764–770. doi:10.1093/bioinformatics/btr011

5. Vurture GW, Sedlazeck FJ, Nattestad M, Underwood CJ, Fang H, Gurtowski J, et al. GenomeScope: fast reference-free genome profiling from short reads. Bioinformatics. 2017;33: 2202–2204. doi:10.1093/bioinformatics/btx153

6. Li H, Durbin R. Fast and accurate short read alignment with Burrows-Wheeler transform. Bioinformatics (Oxford, England). 2009;25: 1754–1760. doi:10.1093/bioinformatics/btp324

7. Li H. Minimap2: pairwise alignment for nucleotide sequences. Birol I, editor. Bioinformatics. 2018;34: 3094–3100. doi:10.1093/bioinformatics/bty191

8. Kajitani R, Toshimoto K, Noguchi H, Toyoda A, Ogura Y, Okuno M, et al. Efficient de novo assembly of highly heterozygous genomes from whole-genome shotgun short reads. Genome Res. 2014;24: 1384–1395. doi:10.1101/gr.170720.113

9. Ye C, Hill CM, Wu S, Ruan J, Ma ZS. DBG2OLC: Efficient assembly of large genomes using long erroneous reads of the third generation sequencing technologies. Sci Rep. 2016;6: 31900. doi:10.1038/srep31900

10. Walker BJ, Abeel T, Shea T, Priest M, Abouelliel A, Sakthikumar S, et al. Pilon: An integrated tool for comprehensive microbial variant detection and genome assembly improvement. PLoS ONE. 2014;9: e112963-14. doi:10.1371/journal.pone.0112963

11. Sahlin K, Vezzi F, Nystedt B, Lundeberg J, Arvestad L. BESST - efficient scaffolding of large fragmented assemblies. BMC bioinform. 2014;15: 281. doi:10.1186/1471-2105-15-281

12. Waterhouse RM, Seppey M, Simão FA, Manni M, Ioannidis P, Klioutchnikov G, et al. BUSCO applications from quality assessments to gene prediction and phylogenomics. Mol Biol Evol. 2017;35: 543–548. doi:10.1093/molbev/msx319

13. Zdobnov EM, Tegenfeldt F, Kuznetsov D, Waterhouse RM, Simão FA, Ioannidis P, et al. OrthoDB v9.1: cataloging evolutionary and functional annotations for animal, fungal, plant, archaeal, bacterial and viral orthologs. Nucleic Acids Res. 2016;45: D744–D749. doi:10.1093/nar/gkw1119

14. Haas BJ, Papanicolaou A, Yassour M, Grabherr M, Blood PD, Bowden J, et al. De novo transcript sequence reconstruction from RNA-seq using the Trinity platform for reference generation and analysis. Nat Protoc. 2013;8: 1494–1512. doi:10.1038/nprot.2013.084

15. Flynn JM, Hubley R, Goubert C, Rosen J, Clark AG, Feschotte C, et al. RepeatModeler2 for automated genomic discovery of transposable element families. Proc National Acad Sci. 2020;117: 9451–9457. doi:10.1073/pnas.1921046117

16. Zhai Z, Nuylert A, Isobe K, Asano Y. Effects of codon optimization and glycosylation on the high-level production of hydroxynitrile lyase from *Chamberlinius hualienensis* in *Pichia pastoris.* J Ind Microbiol Biot. 2019;46: 887–898. doi:10.1007/s10295-019-02162-w
